# From cabinet to PlasmaBag: validating mobile endoscope storage with plasma technology

**DOI:** 10.1128/spectrum.00515-26

**Published:** 2026-08-31

**Authors:** Inès Beaugelin, Caroline Radix, Lionel Pineau

**Affiliations:** 1 Eurofins Biotech-Germande 674549 , Aix en Provence, France; Duke University, Durham, North Carolina, USA

**Keywords:** PlasmaTYPHOON+, patient safety, drying and storage of endoscopes, endoscope reprocessing

## Abstract

This study aimed to assess the microbiological performance of the mobile drying and storage system PlasmaTYPHOON+/PlasmaBAG (Plasmabiotics) by applying the test protocol defined in EN 16442:2015 standard, typically used to validate fixed storage cabinets for endoscopes. A range of endoscopes were artificially contaminated with a suspension of *Pseudomonas aeruginosa* (between 1.5 × 10³ and 5.0 × 10³ CFU/mL). Endoscopes were dried using the PlasmaTYPHOON+ unit and stored in single-use PlasmaBAG for durations ranging from 12 h to 744 h (31 days). Microbiological sampling was performed at each time point using validated recovery methods. Results were compared to positive controls. For all endoscopes dried with PlasmaTYPHOON+ and stored in PlasmaBAGs, the residual contamination index remained negative throughout the 744-h storage period. In contrast, endoscopes stored without drying showed a progressive increase in contamination, reaching up to 5.2 log₁₀ CFU/endoscope. Drying tests confirmed the absence of residual moisture in all channels after treatment with PlasmaTYPHOON+. The application of EN 16442 protocol to this mobile system demonstrated that PlasmaTYPHOON+/PlasmaBAG provides effective drying and long-term microbiological protection of endoscopes. These findings support the extension of EN 16442 performance criteria to mobile storage technologies and validate their integration into routine reprocessing workflows. The system also offers promising applications for the secure transport of endoscopes after maintenance.

**IMPORTANCE:**

Drying and storage are critical steps in endoscope reprocessing, yet they remain insufficiently standardized compared to cleaning and disinfection. Current regulations, such as EN 16442, focus exclusively on fixed storage cabinets, leaving individual storage solutions outside the scope of validation despite their growing use in clinical practice. This study demonstrates that mobile systems combining rapid drying and controlled storage can achieve microbiological safety equivalent to EN 16442 annex E requirements. These findings highlight the need to amend existing standards to include individual storage technologies, ensuring harmonized validation criteria across all modalities. Expanding the regulatory framework will strengthen infection prevention strategies and support the safe integration of innovative solutions into healthcare workflows.

## INTRODUCTION

Outbreaks of endoscope-associated infections resulting in patient deaths have been reported in the literature (1–3). In most reported cases, endoscope-associated infections are linked to lapses in reprocessing procedures and design complexity or damage to the device itself, which may hinder effective decontamination (4, 5).

Among the seven steps of endoscope reprocessing, namely, (i) precleaning, (ii) cleaning, (iii) rinsing, (iv) high-level disinfection, (v) final rinse, (vi) drying, and (vii) storage (6), the last two steps have been neglected for a long time despite their importance in the reprocessing cycle of endoscopes (7). In its 2008 review, Muscarella identified inconsistencies regarding the attention given to drying in different guidelines and in the mind of the hygiene department workers (8). Some guidelines recommend drying of the scope throughout the day and before storage, while some other guidelines recommend drying only before storage (9). According to other published expert opinions, the most commonly observed issues regarding storage are incomplete or absent drying, as well as failure to store the endoscopes in a vertical position (10). Poor drying and storage practices, combined with inadequate cleaning and disinfection, are the main sources of endoscope contamination and possibly post-endoscopic infections (11).

Ineffective drying of endoscope channels can lead to the survival and proliferation of remaining pathogens during storage, as well as promote bacterial growth and biofilm formation due to the presence of contaminated water within the channels (12, 13). Unfortunately, several studies have demonstrated that conventional endoscope drying methods (e.g. filtered compressed air, alcohol flush) were only partially effective in eliminating residual moisture from internal channels (13, 14). Barakat et al. showed that manual drying was less effective than automated systems, and the authors recommended a 10-min drying cycle to increase patient safety by reducing the load of bacteria remaining in the channels (14). Ofstead advocates for stricter automated drying procedures; their “new forced-air drying system” showed significant drying efficacy compared to washer-disinfector alcohol-flush drying systems, which is the standard procedure in the USA (15).

Regarding storage effectiveness, few studies are available (14, 16, 17). AAMI publication from May to June 2020 highlights that storage is no longer just about physical protection, and it must prevent microbial growth and recontamination (18). The authors of the guideline refer the responsibility of drying and storage efficacy to storage cabinet manufacturers using validated protocols (19). In the same way, Grandval et al. (20) demonstrated that when endoscope storage cabinets (ESET) are properly validated according to NF EN 16442:2015, they can effectively maintain the microbiological quality of thermosensitive endoscopes during storage, provided that drying is adequate and cross-contamination is prevented.

In Europe, storage cabinets designed for thermolabile endoscopes must demonstrate their ability to maintain microbiological quality during storage in accordance with the EN 16442:2015 (21) standard, although the drying function remains optional. However, the scope of this standard does not include storage systems using chemical agents, nor individual storage technologies using bags such as the PlasmaTYPHOON+ unit. Among the new technologies, the PlasmaTYPHOON+ system offers an advanced endoscope drying solution that uses a combination of laminar and turbulent heated airflow to rapidly and thoroughly eliminate residual moisture from internal channels, achieving complete drying in under 3 min. Once dried, the endoscope is placed in the PlasmaBAG, a single-use plasma-activated storage solution designed to maintain microbiological integrity for up to 31 days. Together, these components represent an innovative alternative to conventional drying and storage cabinets.

In the absence of a dedicated standard to demonstrate the effectiveness of such products, the French guidelines published in November 2023 by the Ministry of Health and Prevention (13) recommend applying all relevant requirements and associated test protocols from EN 16442 to validate the efficacy of these individual storage units (22).

The aim of this study was to assess whether the EN 16442 test protocol, originally designed for conventional endoscope storage cabinets, can be applied to individual storage systems. Additionally, the study evaluates whether the PlasmaTYPHOON+ system (PLASMABIOTICS) can achieve a level of microbiological safety compared to that required by the standard.

## MATERIALS AND METHODS

### Device description

The PlasmaTYPHOON+ dries endoscopes of major brands within 1–3 min under standardized drying conditions. The system uses two types of airflow to ensure that each individual channel is perfectly dried: a laminar flow to eliminate the residual fluid in the channel and a turbulent heated flow to further and completely dry the channel walls. In combination with complete drying, storage of the endoscope in the single-use PlasmaBAG ensures the preservation of its disinfected state for up to 31 days 1. To provide a safe and active storage environment, the PlasmaBAG is insufflated with plasma containing ozone molecules and then sealed.

### Storage test

A *Pseudomonas aeruginosa* CIP 103467 test suspension containing between 1.5 × 10^3^ and 5.0 × 10^3^ CFU/mL was prepared in a peptone saline diluent (Maximum Recovery Diluent, Oxoid CM0733).

Ultrasound endoscope, gastroscope, and colonoscope were contaminated by injecting 15 mL of the test suspension into endoscope channels. Six milliliters was injected through the suction/instrument channel using a syringe connected to the suction port, 6 mL into the air/water channel via the air connector, and 3 mL through the auxiliary channel connector (if applicable). The bronchoscope was contaminated by injecting 2 mL through the suction port and 2 mL through the biopsy channel inlet.

After 30 min of incubation at room temperature, endoscope channels were purged with 50 mL of air per channel to eliminate the excess of contamination solution. Endoscopes were then maintained at room temperature for 60 min. Endoscopes were then connected to the PlasmaTYPHOON+ drying unit (PLASMABIOTICS) according to the manufacturer’s instructions, using PlasmaTYPHOON+ connectors. Prior to connection, endoscope connectors were dried using an air gun (see drying test), and the surfaces of the PlasmaTYPHOON+ connectors intended to come into contact with the endoscope were disinfected using a wipe pre-impregnated with a detergent-disinfectant solution (Surfa’Safe Premium, ANIOS). After completion of the drying cycle, the endoscope was placed in a PlasmaBAG (PLASMABIOTICS) and stored for 12, 24, 48, 168, 720, and 744 h. After storage, endoscopes were sampled using a sterile sampling solution containing a neutralizer and a surfactant composed of polysorbate 80 (Sigma, P1754) (30 mL), L-histidine (Sigma H-8000) (1 g), lecithin (Acros Organics 413102500) (3 g), and sodium thiosulfate (Sigma 72049-250g) (5 g), in distilled water (QS to 1 L).

Prior to sampling, endoscopes were equipped with specific, clean, and disinfected connectors provided by the endoscope manufacturers.

The sampling protocol described in Annex E of EN 16442 was applied as follows: 50 mL of the sampling solution was injected through the operating channel and 50 mL through the suction channel and recovered at the distal end. Then, one passage of channel brush inside biopsy channel (Tec Care, ENR04-230) was performed. Again, 50 mL of the sampling solution was injected through the operating channel and 50 mL through the suction channel and recovered at the distal end. Fifty milliliters of sampling solution was injected in the air/water channel through the air connector and recovered at the distal end of the endoscope. When an auxiliary channel was present, 20 mL of recovery solution was injected through this channel and recovered at the distal end of the endoscope.

All volumes of sampling solution recovered at the distal end of the endoscope were analyzed for the presence of viable microorganisms, by the pour plate method (1 mL) and by filtration of the remaining volume on 0.45 µm membrane filters (VWR 611-0679). Membrane filters were then incubated on counting agar (Tryptone Soya Agar, Biokar A1401HA) at 37°C ± 1°C for 48 h, as well as the agar plates used for the counting made by dilution/inclusion. For both methods, the counting agar used was Tryptone Soya Agar (Biokar A1401HA). After incubation, colonies were counted, and results were expressed as a number of viable microorganisms per endoscope [*X*_(CFU/endoscope)_].

For the positive controls, the same tests as those described above were performed. However, in this case, endoscopes were stored in a sterile drape without being dried with the PlasmaTYPHOON+ or stored in the PlasmaBAG. The residual contamination index of the endoscopes (*E*_int_) was determined from the difference between the logarithmic value of the number of viable microorganisms recovered from the endoscope channels after drying and storage with the PlasmaTYPHOON+/PlasmaBAG system (*X*_*n*_) and the logarithmic value of the theoretical number of microorganisms present in the endoscope before storage (*B*). The residual contamination index (*E*_int_) was calculated using the following logarithmic formula: *E*_int_ = log_10_(*X*_*n*_) – log_10_(*B*). Negative *E*_int_ values indicate a reduction relative to the theoretical initial burden, whereas positive values indicate an increase.

*B* was calculated from the inoculum density of microorganisms in the test suspension (*N*), the volume of contamination solution injected (*V*), and the recovery performance of the contamination method (*R*) (*B* = *N* × *V* × *R*).

For the PlasmaTYPHOON+/PlasmaBAG storage study, each endoscope model, storage condition, and sampling time point was evaluated in duplicate independent experiments. Results presented in Fig. 1 and 2 are expressed as mean values with standard deviations.

**Fig 1:**
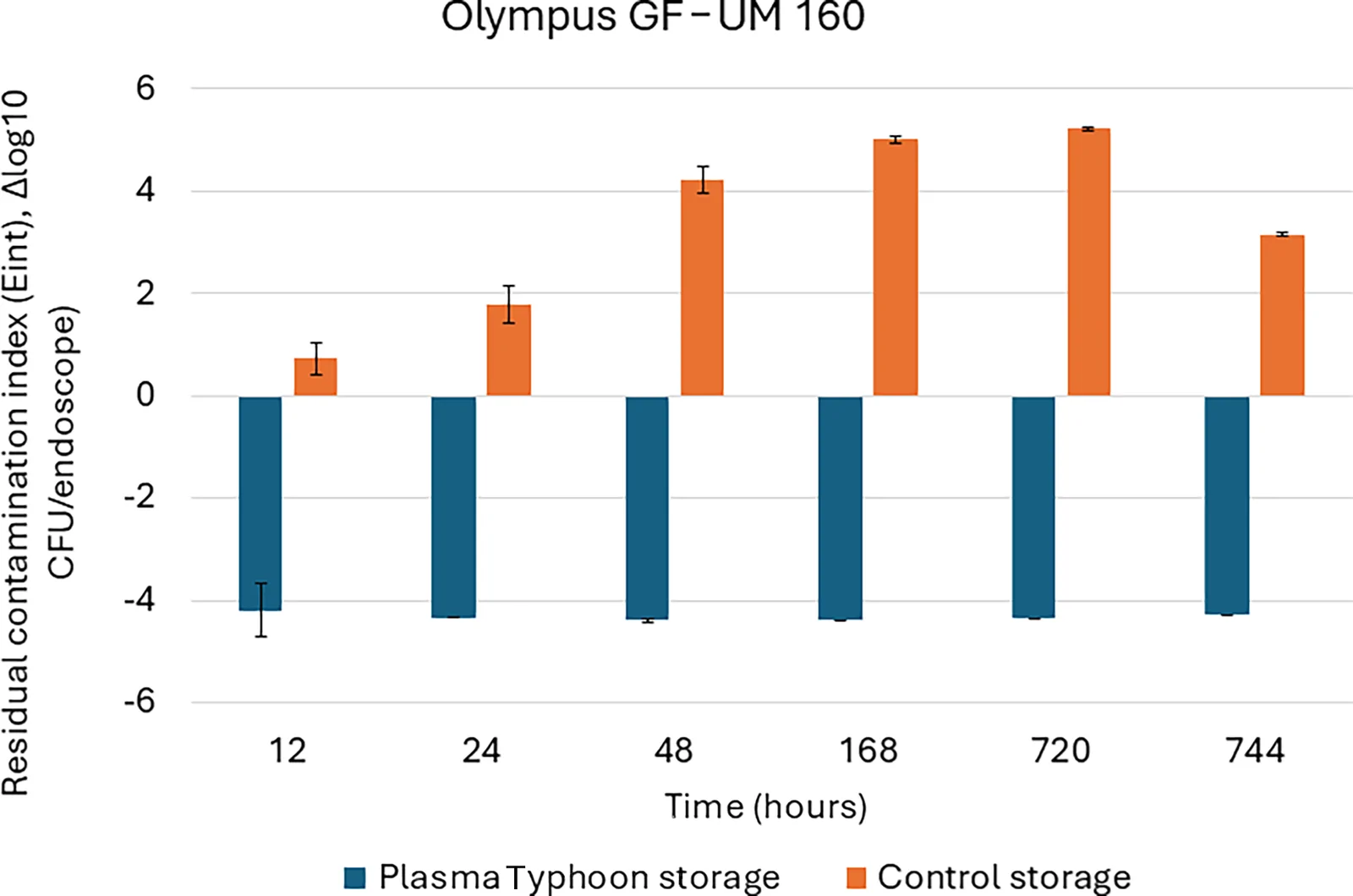
Residual contamination index *E*_int_ = log_10_(*X*_*n*_) – log_10_(*B*) for the ultrasound endoscope OLYMPUS GF-UM160 after PlasmaTYPHOON+ drying followed by PlasmaBAG storage (■) or storage in a sterile drape without treatment (■). Values are mean ± SD from duplicate independent experiments. Negative values indicate a reduction relative to the theoretical initial burden, whereas positive values indicate an increase.

**Fig 2:**
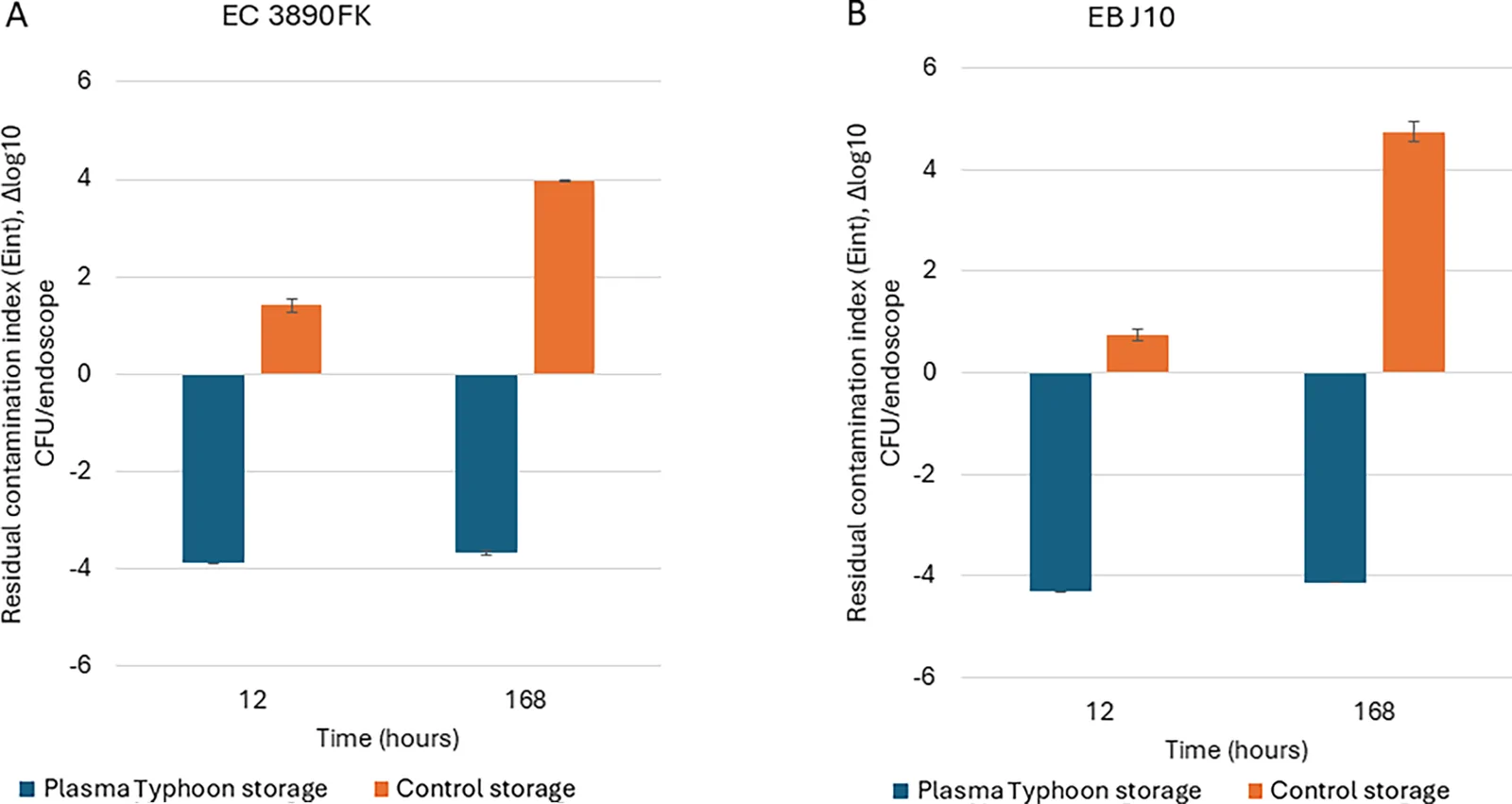
Residual contamination index *E*_int_ = log_10_(*X*_*n*_) – log_10_(*B*) for PENTAX EC3890FK (**A**) and PENTAX EBJ10 (**B**) after PlasmaTYPHOON+ drying followed by PlasmaBAG storage (■) or storage in a sterile drape without treatment (■). Values are mean ± SD from duplicate independent experiments. Negative values indicate a reduction relative to the theoretical initial burden, whereas positive values indicate an increase.

### Recovery ratio

All endoscope test tubes have been subjected to a recovery sampling method validation according to NF EN ISO 11137-1 Annex C (recovery ratio above 80%, data not shown) (23).

### Endoscopes

Tests were performed on a list of endoscopes selected by the manufacturer based on their internal structure and representing different worst-case scenarios: OLYMPUS CF Q180AI colonoscope, OLYMPUS CF-Q160L colonoscope, FUJIFILM EC-600WL colonoscope, FUJIFILM EC-250WM5 colonoscope, PENTAX EC-380MK2P colonoscope, PENTAX ES-3870K sigmoidoscope, OLYMPUS GIF-Q160 gastroscope, OLYMPUS GIF-Q180 gastroscope, OLYMPUS GF-UM160 ultrasound scope, and PENTAX FCN-15X bronchoscope. Between tests, endoscopes were reprocessed using a semi-automatic cleaning-disinfection procedure.

### Drying test

As specified by the manufacturer of the drying unit PlasmaTYPHOON+, the air/water and the suction valve housings located on the endoscope control handle, the air pipe and the water bottle connector, the suction connector, and the biopsy channel inlet port were dried with an air gun before connecting the endoscope to the PlasmaTYPHOON+ unit. Once the endoscope’s channels were connected to the drying unit PlasmaTYPHOON+, a drying process was initiated according to the manufacturer’s instructions (PLASMABIOTICS). According to the EN 16442 (21), once the drying process was finished, compressed medical-grade air at a positive pressure up to 120 kPa was blown through each channel of the endoscopes, with the distal end of the endoscope positioned between 50 mm and 100 mm above and perpendicular to a colored test paper. The test was compliant if no trace of moisture was detected on the paper. Any color change of the test paper was considered evidence of residual moisture.

### Statistical analysis

Microbiological results were expressed using the residual contamination index *E*_int_ = log_10_(*X*_*n*_) − log_10_(*B*), where *X*_*n*_ is the number of viable microorganisms recovered from the endoscope after storage and *B* is the theoretical initial microbial burden introduced before storage. For each endoscope model, storage condition, and time point, assays were performed in duplicate independent experiments, and results are reported as mean ± standard deviation (SD). Because the number of independent experiments per condition/time point was limited (*n* = 2), statistical analysis was primarily descriptive. As an exploratory non-parametric analysis, matched comparisons between PlasmaTYPHOON+/PlasmaBAG storage and sterile drape control storage were assessed across paired scope/time-point mean *E*_int_ values using a two-sided exact sign test. For each matched pair, the difference was defined as mean *E*_int_(control) − mean *E*_int_(PlasmaTYPHOON+/PlasmaBAG); positive values therefore indicated lower residual contamination in the PlasmaTYPHOON+/PlasmaBAG condition. Pairs with zero difference would have been excluded from the sign test; however, no zero differences were observed.

## RESULTS

Figure 1 shows the change in recovered microbial burden relative to the theoretical initial burden (*E*_int_) for the ultrasound endoscope OLYMPUS GF-UM160. Across the independent experimental runs, the inoculum density used to contaminate this endoscope ranged from approximately 2.1 × 10^3^ to 3.9 × 10^3^ CFU/mL. In the control condition, i.e., storage in a sterile drape without PlasmaTYPHOON+ drying and without PlasmaBAG storage, the mean *E*_int_ increased over time from 0.72 ± 0.33 log_10_ after 12 h to 5.21 ± 0.04 log_10_ after 720 h, before decreasing to 3.15 ± 0.02 log_10_ after 744 h.

In contrast, when the same endoscope was dried using the PlasmaTYPHOON+ system and stored in a PlasmaBAG, an immediate reduction in intraluminal bacterial burden was observed—approximately a 4 log₁₀ decrease relative to the initial contamination level after only 12 h of storage. No subsequent increase in contamination was observed for the duration of the storage period, up to 744 h.

A similar pattern was observed for the PENTAX EC3890FK and PENTAX EBJ10 endoscopes (Fig. 2). After PlasmaTYPHOON+ drying followed by PlasmaBAG storage, mean *E*_int_ values remained negative at both sampling times (12 h and 168 h) for both endoscope models (EC3890FK: −3.89 ± 0.00 and −3.68 ± 0.04 log_10_; EBJ10: −4.31 ± 0.00 and −4.13 ± 0.00 log_10_). In contrast, in the sterile drape control condition, mean *E*_int_ values were positive and increased over time (EC3890FK: 1.41 ± 0.14 at 12 h and 3.98 ± 0.00 log_10_ at 168 h; EBJ10: 0.75 ± 0.11 at 12 h and 4.74 ± 0.19 log_10_ at 168 h).

Across the 10 matched scope/time-point comparisons available in Fig. 1 and 2, mean *E*_int_ values were lower after PlasmaTYPHOON+/PlasmaBAG storage than after sterile drape control storage in every case. This consistent direction of effect was supported by the exploratory two-sided exact sign test (10/10 comparisons in favor of PlasmaTYPHOON+/PlasmaBAG; *P* = 0.00195).

The results of the drying tests are presented in Table 1. For all the endoscopes tested, no traces of moisture have been detected in the endoscopes after drying with the PlasmaTYPHOON+.

**TABLE 1:** Results of drying tests performed on endoscope

Brand	Type	Cycle of PlasmaTYPHOON+ tested	Results
OLYMPUS	CF-Q180AI	Digestive endoscopes	No traces of moisture
OLYMPUS	CF-Q160L		No traces of moisture
FUJIFILM	EC-600WL	Bronchoscope	No traces of moisture
FUJIFILM	EC-250WM5		No traces of moisture
PENTAX	EC-380MK2P		No traces of moisture
PENTAX	ES-3870K		No traces of moisture
OLYMPUS	GIF-Q160		No traces of moisture
OLYMPUS	GIF-Q180		No traces of moisture
OLYMPUS	GF-UM160		No traces of moisture
PENTAX	FCN-15X		No traces of moisture

## DISCUSSION

The EN 16442 standard was developed to evaluate the drying and storage performance of fixed endoscope storage cabinets, with a focus on ensuring microbiological safety through effective moisture removal and contamination control (21). However, the increasing need for flexible, mobile, and space-efficient storage solutions has led to the emergence of individual storage systems such as endoscope storage bags. Despite their growing use, these systems have not been systematically validated under the same rigorous framework as fixed cabinets. Considering that both fixed storage cabinets and mobile individual storage systems share the same fundamental objective—namely, ensuring effective endoscope drying and maintaining microbiological integrity during storage—it is reasonable to propose that the performance requirements defined in the EN 16442 standard should be equally applicable to both formats. The core principles of drying efficiency and contamination prevention are universal, regardless of whether the system is stationary or mobile. Extending the scope of EN 16442 to include mobile storage solutions would therefore promote harmonized safety and quality standards across all endoscope storage modalities. This position is consistent with recent French guidelines (FAQ no. 3, 2023) (22), which advocate for the evaluation of individual storage systems using the same criteria applied to fixed cabinets. Our findings support this approach, as the successful application of EN 16442 to the PlasmaTYPHOON+ system demonstrates that the main tests of EN 16442 can be used to evaluate mobile storage technologies.

Performance tests were conducted in this study by adapting Annex E of the EN 16442 standard. Annex E outlines a standardized microbiological test protocol to verify that endoscopes stored in a cabinet do not exhibit increased internal contamination over time, ensuring that the system maintains microbiological integrity during storage. We have demonstrated that PlasmaTYPHOON+/PlasmaBAG combined process not only achieves effective drying across a wide range of endoscope models but also maintains microbiological integrity during extended storage periods—up to 744 h—without any increase in contamination levels. The results obtained in this study are comparable to those reported by Pineau et al. (24), who demonstrated the effectiveness of a drying and storage cabinet in reducing microbial contamination in endoscope channels during storage. In their study, endoscopes stored in a controlled cabinet environment showed a significant reduction in bacterial load—up to 4.6 log₁₀ units—while those stored in uncontrolled conditions exhibited stable or increasing contamination levels. Although some differences in absolute values may be observed—likely due to variations in storage configuration, such as the vertical positioning of endoscopes in Pineau’s study—the overall trend remains consistent. These findings reinforce the conclusion that controlled drying and storage environments, whether fixed or mobile, play a critical role in preserving the microbiological integrity of endoscopes between uses (20). This interpretation is further supported by the exploratory exact sign test, which showed that the direction of the effect consistently favored PlasmaTYPHOON+/PlasmaBAG storage across all matched scope/time-point comparisons.

Other recent studies have highlighted the effectiveness of the PlasmaTYPHOON+ system as an innovative solution for the rapid drying and active storage of heat-sensitive endoscopes. In a clinical simulation study conducted at Erasmus Medical Center 5, the system demonstrated a significant reduction in microbial contamination following standard reprocessing, with complete elimination of detectable contamination in two out of three duodenoscopes and a marked decrease in residual moisture levels (17). These findings support the system’s ability to prevent microbial proliferation by ensuring rapid and thorough internal drying. Furthermore, large-scale clinical evaluations, such as those conducted at the University Hospital Frankfurt, confirmed the system’s ability to maintain microbiological integrity of endoscopes for up to 31 days when combined with the PlasmaBAG storage solution (25). Compared to conventional drying cabinets and morning reprocessing protocols, the PlasmaTYPHOON+ offers additional benefits, including reduced energy and consumable use, enhanced traceability, and improved workflow efficiency. These data support the integration of PlasmaTYPHOON+ as a reliable alternative to traditional storage methods, particularly in high-throughput endoscopy units where rapid turnaround and infection prevention are critical.

Beyond routine clinical use, mobile storage systems also offer promising applications in the management of endoscopes returning from external maintenance or repair. Given the lack of control over environmental conditions during transport, many national and international guidelines currently recommend microbiological sampling of endoscopes upon receipt, prior to reuse. However, the emergence of validated mobile storage and transport systems capable of maintaining controlled microbiological conditions throughout transit could eliminate the need for such systematic post-transport verification. Provided that microbiological integrity is confirmed before dispatch and maintained during transport, these systems could ensure that endoscopes remain compliant with hygiene standards upon arrival. As highlighted in the latest French Q&A (November 2023), this approach requires clear protocols for verifying the integrity of transport containers and for documenting microbial control throughout the transport process—offering a significant opportunity to streamline workflows while maintaining patient safety.

This study has some limitations. First, the experimental design was primarily intended as a descriptive adaptation of the EN 16442 Annex E validation framework and was not powered for extensive inferential statistical comparisons. Although each condition and time point was assessed in duplicate independent experiments, larger numbers of replicates would be required to more precisely quantify inter-run variability and to support more complex statistical modeling. In addition, the exploratory exact sign test used here only assesses the consistency of the direction of the observed effect across matched comparisons and does not take into account the magnitude of the differences. However, the consistency of the observed trends across all endoscope models and storage durations strengthens the overall interpretation of the data.

In summary, this study demonstrates that the PlasmaTYPHOON+ system, despite its fundamentally different design from traditional fixed storage cabinets, delivers comparable performance in drying efficiency and microbiological preservation. The successful adaptation of the EN 16442 testing protocol to this mobile system provides strong evidence of its reliability and supports its integration into routine endoscope reprocessing workflows. These findings advocate for an expanded interpretation of EN 16442, encompassing innovative mobile storage technologies and promoting harmonized safety standards across all storage modalities.
